# Nigrostriatal dopaminergic vulnerability in Parkinson’s disease: Neuroprotective strategies

**DOI:** 10.4103/NRR.NRR-D-25-00380

**Published:** 2025-08-13

**Authors:** Estefanía Santana-Román, Luis O. Soto-Rojas, Elias Manjarrez, Oscar Arias-Carrión

**Affiliations:** 1 División de Neurociencias, Clínica, Instituto Nacional de Rehabilitación Luis Guillermo Ibarra Ibarra, Mexico City, Mexico; 2 Laboratorio de Patogénesis Molecular, Facultad de Estudios Superiores Iztacala, Universidad Nacional Autónoma de México, Ciudad de México, México; 3 Instituto de Fisiología, Benemérita Universidad Autónoma de Puebla, Puebla, México

**Keywords:** dopamine metabolism, dopaminergic neurons, iron accumulation, neurodegeneration, neurovascular factors, nigrostriatal pathway, oxidative stress, Parkinson’s disease, plasticity, α-synuclein

## Abstract

The selective vulnerability of nigrostriatal dopaminergic neurons is a hallmark of Parkinson’s disease and underlies its progressive motor decline. These neurons are uniquely susceptible to degeneration due to their extensive axonal arborization, high energy demands, sustained pacemaking activity, and cytosolic dopamine metabolism, which collectively promote oxidative stress and mitochondrial dysfunction. Advances in single-nucleus RNA sequencing and spatial transcriptomics have revealed transcriptionally distinct dopaminergic subtypes within the human substantia nigra pars compacta, such as AGTR1^+^/SOX6^+^ and RIT2^+^ populations, which exhibit subtype-specific transcriptional stress signatures and are preferentially lost in Parkinson’s disease. These findings underscore the role of intrinsic vulnerability, influenced by genetic risk loci, mitochondrial stress, and protein misfolding pathways, including α-synuclein aggregation. Furthermore, neuroinflammation, iron accumulation, and vascular dysfunction act synergistically to amplify neuronal loss. This review integrates molecular, cellular, and systems-level mechanisms contributing to dopaminergic degeneration and evaluates emerging neuroprotective strategies. These include anti-oxidative, anti-inflammatory, mitochondrial therapies, novel biomarkers, gene editing, and cell replacement techniques. Understanding the selective vulnerability of nigrostriatal subtypes offers a promising path toward precision-targeted, disease-modifying treatments for Parkinson’s disease.

## Introduction

The discovery of dopamine’s role as a neurotransmitter in the late 1950s transformed the understanding of neurological disorders, particularly Parkinson’s disease (PD). Using histofluorescence techniques, Carlsson and colleagues first identified distinct dopaminergic neuronal populations (Carlsson et al., 1957), and soon after, Birkmayer and Hornykiewicz (1961) demonstrated that PD was characterized by striatal dopamine deficiency, with levodopa therapy producing symptomatic relief. A subsequent histological study by Dahlström and Fuxe (1964) confirmed that degeneration of neurons in the substantia nigra pars compacta (SNpc) was a pathological hallmark of PD, correlating closely with the emergence of motor symptoms. These foundational discoveries established the centrality of nigrostriatal dopamine loss in PD pathophysiology, guiding decades of neurochemical, electrophysiological, and pharmacological research (Hornykiewicz, 1998, 2006).

Although dopaminergic depletion underlies the hallmark motor features of PD, the disorder is now recognized as a complex, multisystem neurodegenerative condition. Neurodegeneration extends beyond the nigrostriatal pathway, affecting noradrenergic neurons in the locus coeruleus, cholinergic cells in the basal forebrain, and serotonergic and glutamatergic circuits, contributing to non-motor symptoms such as cognitive impairment, sleep disturbances, and mood disorders (Yahr and Duvoisin, 1972; Paredes-Rodriguez et al., 2020). Despite this broader neurodegeneration, the selective vulnerability of SNpc dopaminergic neurons remains a defining and incompletely understood feature of PD (Marsden and Parkes, 1977; de Rijk et al., 2000).

Nigrostriatal dopaminergic neurons are exposed to distinct metabolic, physiological, and structural stressors that may render them particularly susceptible to degeneration. Their vast axonal arborization and high synaptic burden necessitate elevated energy consumption, making them disproportionately vulnerable to mitochondrial dysfunction and oxidative stress (O’Callaghan et al., 2021). These neurons also maintain sustained pacemaker activity with calcium influx, which compounds mitochondrial strain and accelerates cellular aging (Bohnen et al., 2022) at the molecular level, converging insults from α-synuclein aggregation, proteostatic collapse, and neuroinflammatory signaling cascade into progressive neuronal damage, distinguishing SNpc neurons from more resilient neighboring populations, such as those in the ventral tegmental area (VTA) (Braak and Del Tredici, 2008; Bohnen et al., 2022).

A longstanding debate in PD pathogenesis concerns whether this selective vulnerability arises primarily from intrinsic factors, such as dopamine metabolism, mitochondrial inefficiency, and lysosomal burden, or extrinsic processes, including α-synuclein propagation, neuroinflammation, and environmental toxins. According to Braak’s staging model, PD pathology originates in the gut and olfactory bulb and spreads transneuronally to the brainstem and midbrain via the vagus nerve (Braak et al., 2003; Paredes-Rodriguez et al., 2020). However, alternative hypotheses emphasize intrinsic cellular stress and transcriptional dysregulation within SNpc neurons as early drivers of neurodegeneration (Chaudhuri et al., 2006; Garcia-Ruiz et al., 2014).

Recent advances using multi-omic approaches, such as single-nucleus RNA sequencing (snRNA-seq) and spatial transcriptomics, have provided compelling evidence for transcriptionally defined dopaminergic neuron subtypes within the human SNpc, each displaying differential vulnerability to PD (Kamath et al., 2022; Wang et al., 2024). Notably, a ventrally localized population co-expressing AGTR1 and SOX6 was found to be selectively depleted in PD, with transcriptomic signatures enriched in degeneration-associated pathways, including TP53 and NR2F2 signaling (Kamath et al., 2022). These populations also showed high overlap with PD-associated risk loci identified by genome-wide association studies, reinforcing that cell-intrinsic properties may determine neuron fate. Complementary studies using spatial transcriptomics confirmed that RIT2-expressing subtypes — implicated in dopamine signaling and vesicular transport — were also selectively lost in PD, further refining the concept of dorsoventral vulnerability in human SNpc (Wang et al., 2024).

Such findings underscore the need for human-specific molecular atlases and caution against overreliance on rodent models, which lack specific vulnerable subtypes such as CALB1_GEM neurons (Kamath et al., 2022). Moreover, transcriptional regulators such as NURR1, FOXA1/2, and PITX3, essential for dopaminergic differentiation and mitochondrial integrity, may orchestrate susceptibility by governing developmental lineage and stress responses (Hook et al., 2018; Carmichael et al., 2021). These insights immediately impact precision biomarker development and targeted neuroprotective strategies to preserve the most vulnerable neuronal subtypes.

Understanding the mechanisms of selective vulnerability in PD, integrating cellular, molecular, and circuit-level data, is vital for advancing early diagnosis and disease-modifying therapies. This review synthesizes current findings on intrinsic and extrinsic mechanisms of dopaminergic degeneration, emphasizing molecular subtypes, and highlights therapeutic strategies that may preserve neuron integrity and restore functional networks in PD.

## Data Sources

A structured search strategy was applied across PubMed, Scopus, and the Web of Science databases to ensure a comprehensive and up-to-date synthesis of the literature on nigrostriatal vulnerability and neuroprotection in PD. The search was conducted between January 2023 and March 2025 and included a combination of Medical Subject Headings (MeSH) and free-text terms. Boolean operators (AND, OR, NOT) were used to refine search outputs and maximize the retrieval of relevant, high-quality studies.

The primary search terms included: “nigrostriatal pathway, dopaminergic neurons, Parkinson’s disease, oxidative stress, mitochondrial dysfunction, dopamine metabolism, α-synuclein, iron accumulation, neuroinflammation, microglia, astrocytes, selective vulnerability, single-cell RNA sequencing, neurodegeneration, neuroprotection, and transcriptomic profiling.” Recent focus areas, such as spatial transcriptomics, inflammasome signaling, and cell subtype–specific degeneration, were specifically targeted to reflect the evolving landscape of PD research.

Inclusion criteria were restricted to peer-reviewed articles published in English that Investigated the molecular, cellular, and systems-level mechanisms contributing to the selective vulnerability of nigrostriatal dopaminergic neurons in PD; Presented original data or comprehensive reviews on neurodegenerative mechanisms relevant to dopaminergic circuits; Explored novel or emerging therapeutic strategies, including antioxidant interventions, mitochondrial modulators, immunomodulatory therapies, and neurorestorative techniques (e.g., gene editing, cell-based therapies); Employed advanced techniques such as single-nucleus RNA sequencing, spatial transcriptomics, CRISPR-Cas9, or *in vivo* imaging.

Exclusion criteria included studies not directly related to Parkinson’s disease or dopaminergic neurodegeneration; reports lacking mechanistic insight into dopaminergic vulnerability or neuroprotective intervention; non-peer-reviewed material, editorials, commentaries, or conference abstracts. Both seminal works — including foundational studies on dopamine neurobiology and early histopathological observations — and state-of-the-art publications from 2018 to 2025 were incorporated to ensure historical depth and scientific relevance. Special emphasis was placed on integrative analyses from multi-omic studies that redefine dopaminergic subtypes, developmental lineage, and transcriptional determinants of vulnerability.

This dual approach, combining historical perspective with cutting-edge molecular neuroscience, supports a nuanced review of dopaminergic neuron vulnerability and informs current and future strategies for neuroprotection in PD.

## Nigrostriatal Dopaminergic System

The nigrostriatal dopaminergic system originates from neurons in the SNpc, a pigmented, neuromelanin-rich structure in the midbrain. These neurons project via the medial forebrain bundle to form dense and functionally critical synaptic networks within the striatum, comprising the caudate nucleus and putamen (Arias-Carrion and Poppel, 2007; Arias-Carrion et al., 2010). Despite its small volume — estimated at only 0.056 cm³ — the SNpc exerts synaptic control over the striatum, which is nearly 100 times larger (von Ziegler et al., 2013). This stark volumetric contrast highlights a key pathological vulnerability: damage to the compact SNpc can cause disproportionately widespread dysfunction in basal ganglia circuitry.

Dopaminergic neurons in this system are highly specialized, with extensive axonal arborization and metabolic demands. In rodents, a single nigrostriatal neuron may extend axons up to 800 mm, forming nearly one million synapses and innervating up to 5% of the 2.8 million striatal neurons (Matsuda et al., 2009). This expansive projection network underscores their importance in modulating motor, cognitive, and affective functions but also renders them susceptible to neurodegenerative stressors.

Initial characterizations by Dahlstroem and Fuxe (1964) and later by Ungerstedt delineated the A9 dopaminergic cell group as the origin of these projections. However, molecular and spatial profiling over the past two decades has revealed a far more diverse system. snRNA-seq has identified ten molecularly distinct dopamine (DA) subtypes in the human SNpc (Kamath et al., 2022). One population, co-expressing SOX6 and AGTR1, is preferentially located in the ventral SNpc and is markedly reduced in PD. This vulnerable subtype exhibits a transcriptional profile enriched for PD-associated risk loci and upregulation of pro-degenerative programs involving TP53 and NR2F2 (Kamath et al., 2022).

Complementary evidence from spatial transcriptomic profiling confirms the selective degeneration of RIT2- and SOX6-expressing neurons in PD, alongside reductions in ALDH1A1-positive populations (Wang et al., 2024). These findings align with earlier observations that SV2C, a vesicular protein that enhances dopamine storage, modulates susceptibility to dopaminergic toxins such as 1-methyl-4-phenyl-1,2,3,6-tetrahydropyridine (MPTP) (Brichta and Greengard, 2014). The loss of SV2C-expressing neurons in PD further highlights the importance of subtype-specific molecular composition in disease progression.

These subtypes are defined by neurotransmitter identity and transcription factors essential for dopaminergic differentiation and survival. Key regulators include NURR1, FOXA1/2, and PITX3 (Carmichael et al., 2021). In addition, genes responsible for dopamine synthesis (tyrosine hydroxylase), transport (dopamine transporter [DAT]; vesicular monoamine transporter 2 [VMAT2]), and degradation (ALDH1A1) help demarcate distinct functional and anatomical profiles (Hook et al., 2018).

Interestingly, some of these molecularly defined subtypes, such as the CALB1_GEM population, do not have clear homologs in rodents, underscoring the importance of human-specific models for accurate mechanistic insight (Kamath et al., 2022). This distinction has major implications for therapeutic translation, particularly as these vulnerable subpopulations exhibit mitochondrial and lysosomal stress signatures before overt cell loss, suggesting early intervention windows (Surmeier et al., 2010; Mosharov, 2022).

Moreover, the conventional anatomical boundaries between SNpc and adjacent regions, such as the substantia nigra pars reticulata (SNpr), retrorubral area (A8), and VTA (A10), are functionally permeable. Dopaminergic projections from these neighboring regions selectively target striosome or matrix compartments of the striatum, contributing to the complexity of the so-called mesostriatal system (Poulin et al., 2014; Menegas et al., 2015). This revised view of nigrostriatal architecture, refined by multi-omic technologies such as spatial transcriptomics and epigenomic profiling, provides a framework to understand regional vulnerability better and inform biomarker-guided, subtype-specific therapies in PD (Welch et al., 2021; Wang et al., 2024).

### Dopaminergic neuron subtypes and selective vulnerability in Parkinson’s disease

DA neurons of the midbrain have classically been organized into three anatomically defined regions: the VTA, substantia nigra pars compacta (SNc), and the retrorubral field (Bjorklund and Dunnett, 2007; Fu et al., 2012). However, this anatomical framework is now recognized as insufficient to capture these neurons’ molecular, functional, and pathological diversity. Recent studies have demonstrated that DA neurons within these regions exhibit striking heterogeneity, with distinct subpopulations characterized by divergent electrophysiological signatures, behavioral responses, projection patterns, and disease susceptibilities (Menegas et al., 2015; Evans et al., 2017; Avvisati et al., 2024).

The development of single-cell and single-nucleus transcriptomic technologies has enabled the identification of transcriptionally distinct DA neuron subtypes, especially within the human SNc. Using high-resolution approaches such as snRNA-seq, up to ten molecularly defined DA subtypes have been delineated, each with a unique spatial distribution and gene expression profile (Kamath et al., 2022). Notably, a ventrally localized population co-expressing SOX6 and AGTR1 has emerged as a key site of vulnerability in PD. This population is significantly depleted in postmortem PD brains and shows transcriptional enrichment for PD-associated risk loci and upregulation of degeneration-linked pathways involving TP53 and NR2F2 (Kamath et al., 2022). These findings suggest that cell-intrinsic mechanisms may play a central role in determining neuronal fate in PD.

Complementary data from large-scale profiling of over 300,000 nuclei from human substantia nigra tissues confirmed these observations and further identified DA subtypes, marked by tyrosine hydroxylase (TH), SLC18A2, and SOX6 expression, as preferentially lost in PD (Wang et al., 2024). Interestingly, another subtype enriched for RIT2, a gene implicated in PD genetics, also displayed selective vulnerability, reinforcing the link between subtype-specific gene expression and neurodegeneration (Wang et al., 2024). Importantly, these transcriptional patterns align with neurodevelopmental trajectories shaped by key transcription factors such as LMX1A, OTX2, and PBX1 (Hook et al., 2018). However, the persistence of overlapping transcription factor expression across subtypes suggests a more complex combinatorial or epigenetic regulation of DA neuron identity and function.

Recent findings in non-human primates further support this molecular stratification. In the MPTP monkey model of PD, Del Rey et al. (2024) demonstrated that DA neurons expressing aldehyde dehydrogenase 1 family member A1 (Aldh1a1) and G-protein inward rectifier potassium channel 2 (Girk2) in the ventral SNc are highly vulnerable to degeneration. In contrast, calbindin-expressing neurons in the dorsal SNc and VTA are relatively spared (Del Rey et al., 2024). Aldh1a1⁺/Girk2⁺ neurons were shown to project specifically to the putamen and caudate (nigrostriatal pathway), while Aldh1a1⁻ neurons, largely projecting to the globus pallidus, were preserved. Notably, degeneration of Aldh1a1⁺ neurons and their dendritic bundles, which co-localize with cannabinoid receptor 1 afferents in the SNr, strongly correlated with the onset and severity of parkinsonian symptoms. These anatomical and molecular findings align closely with human data and reinforce that projection patterns, intrinsic molecular profiles, and afferent inputs contribute to subtype-specific vulnerability.

Parallel to these neurocentric models, emerging evidence highlights a critical role for the adaptive immune system in PD pathogenesis. Using large-scale single-cell transcriptomics and T cell receptor sequencing, Wang et al. (2021) profiled over 100,000 T cells from the blood and cerebrospinal fluid of PD patients and healthy controls. They identified clonally expanded cytotoxic CD8^+^ T cells with transcriptional signatures of terminal effector differentiation and a novel population of cytotoxic CD4^+^ T cells derived from Th1-like precursors. These expanded populations expressed high levels of genes associated with cytotoxicity (GZMB and PRF1), adhesion (ITGAM and ITGB1), and migration (CX3CR1 and SELPLG), suggesting their capacity to penetrate the blood–brain barrier and contribute to neuroinflammation. Notably, T cell receptor-based tracking revealed that some clonotypes were shared between the blood and cerebrospinal fluid, implicating peripheral T cells in central immune responses. Computational predictions identified α-synuclein-derived epitopes and MHC alleles likely to drive T cell responses, supporting the hypothesis of antigen-specific T cell–mediated neurodegeneration in PD.

Rodent models, which have been fundamental to dissecting dopaminergic circuits, lack clear homologs for specific human DA subtypes, such as CALB1_GEM neurons. This discrepancy emphasizes the need for human-specific atlases and functional studies to ensure accurate disease modeling and translational relevance (Kamath et al., 2022).

Studying genetic forms of PD, particularly those involving leucine-rich repeat kinase 2 (LRRK2), offers valuable insights. LRRK2 mutations such as G2019S lead to increased kinase activity and widespread disruptions in synaptic vesicle cycling, endocytosis, and cytoskeletal dynamics—essential for DA neuron function. Knock-in mouse models expressing LRRK2 G2019S at physiological levels exhibit impaired DA transmission and subtle circuit-level dysfunction prior to neurodegeneration (Yue et al., 2015; Xenias et al., 2022; Khan et al., 2024). Nevertheless, the cell-autonomous effects of LRRK2 within specific human DA neuron subtypes, particularly those most vulnerable in PD, remain incompletely understood.

The emergence of spatially resolved and integrative multi-omic technologies, such as Slide-seq and LIGER, offers powerful tools to interrogate gene regulatory dynamics, epigenetic landscapes, and spatial contexts of vulnerable neuronal populations (Welch et al., 2021). These approaches are essential for mapping disease progression and identifying subtype-specific therapeutic targets and biomarker candidates.

These findings argue for a paradigm shift in conceptualizing midbrain DA neurons from rigid anatomical groupings to dynamic molecular entities shaped by transcriptional, epigenetic, and spatial features. This redefinition is crucial for understanding the selective vulnerability observed in PD and developing precision strategies aimed at neuroprotection and disease modification.

### Electrophysiological characteristics

Midbrain dopaminergic neurons within the SNpc exhibit distinctive electrophysiological signatures that underpin their function in modulating basal ganglia output and motor control. These neurons maintain a tonic, low-frequency autonomous firing pattern typically ranging from 0.5 to 10 Hz, which ensures stable dopamine release and tonic modulation of striatal targets (Grace and Bunney, 1984). Bursting patterns, facilitated by gap junction-mediated coupling and intrinsic ion channel dynamics, transiently elevate extracellular dopamine levels and are central to behavioral flexibility and reward learning (Chiodo and Bunney, 1985; Beaulieu and Gainetdinov, 2011).

A key physiological constraint of these neurons is the extended refractory period governed by calcium-activated potassium currents, which limit maximal firing rates and influence their susceptibility to neurodegeneration under sustained metabolic stress (Morikawa and Paladini, 2011). This intrinsic property may be therapeutically exploitable: pharmacological or neuromodulatory modulation of ion channel dynamics could help stabilize dopaminergic firing and enhance therapeutic efficacy.

Electrophysiological studies have also shown a dichotomy in activity between dopaminergic and GABAergic populations in the substantia nigra. In response to behavioral feedback, SNpc dopaminergic neurons exhibit brief phasic increases (250–500 ms), whereas GABAergic neurons in the SNpr display delayed and prolonged activation (Ramayya et al., 2014). This temporal dissociation provides insight into basal ganglia output regulation and offers a potential framework for interpreting abnormal circuit dynamics in PD.

*In vivo*, electrophysiological recordings and functional imaging advances have highlighted aberrant network-level synchronization in PD. Excessive beta-band oscillations (13–30 Hz) in the subthalamic nucleus and cortex are consistently observed in dopamine-depleted states and correlate with bradykinesia and rigidity (Lee et al., 2023). These pathophysiological oscillations reflect a breakdown in cortico-subcortical re-entrant loops and offer a biomarker for disease monitoring and neuromodulation (Dirkx and Bologna, 2022).

Deep brain stimulation (DBS), particularly targeting the subthalamic nucleus or globus pallidus internus, has leveraged these electrophysiological insights to suppress pathological oscillations and restore motor function in patients with advanced PD (Hariz and Blomstedt, 2022; Martinez-Nunez and LeWitt, 2023). High-frequency stimulation modulates the balance between excitatory and inhibitory inputs within basal ganglia–thalamocortical circuits, dampening pathological synchrony without necessarily altering average firing rates (Lee et al., 2023).

Recent innovations have shifted DBS from an empirical to a data-driven discipline. Machine learning models based on functional MRI have successfully predicted optimal DBS parameters by mapping stimulation-induced brain activation patterns to motor outcomes (Beaulieu and Gainetdinov, 2011). Automated algorithms such as StimFit and Illumina 3D now integrate patient-specific neuroimaging to identify optimal electrode configurations, improving clinical programming and reducing side effects (Malekmohammadi et al., 2022; Roediger et al., 2022).

Furthermore, emerging studies demonstrate that distinct symptom domains — tremor, bradykinesia, rigidity, and axial disturbances — are modulated by stimulation of separate white matter tracts (Rajamani et al., 2024). This symptom-network specificity supports a shift toward personalized neuromodulation guided by electrophysiological phenotyping and imaging biomarkers.

Despite these advances, translating electrophysiological findings into routine clinical diagnostics remains challenging. Nonetheless, integrating electrophysiological signatures with neuroimaging and genetic markers holds promise for precision therapies. For example, patients with predominant burst-firing pathophysiology may respond preferentially to pulse-patterned DBS or targeted pharmacological modulation, while those with tonic hypoactivity may benefit from network activation strategies. Longitudinal neurophysiological monitoring could serve as a biomarker of disease progression and compensatory adaptation, offering a window into the timing and durability of therapeutic responses.

Ongoing efforts to refine these tools, including closed-loop stimulation systems that adapt to real-time neurophysiological changes, are expected to enhance further the efficacy and safety of neuromodulatory therapies in PD (Hariz and Blomstedt, 2022; Petersen et al., 2022).

### Neurochemical characteristics

DA, a catecholamine synthesized predominantly in the SNpc, plays a central role in motor control, motivation, and behavioral regulation. It exerts its effects through G-protein-coupled receptors, subdivided into D1-like (D1, D5) and D2-like (D2, D3, D4) families, which are unevenly distributed across basal ganglia structures. This heterogeneous receptor topology underlies distinct physiological responses and pharmacological effects in Parkinson’s disease (Beaulieu and Gainetdinov, 2011; Channer et al., 2023; Bhatia et al., 2025).

Dopamine biosynthesis begins with the active transport of tyrosine across the blood–brain barrier, where it is converted to levodopa (L-DOPA) by TH, the rate-limiting enzyme in the dopaminergic pathway. L-DOPA is then decarboxylated to dopamine by aromatic L-amino acid decarboxylase (AADC). Notably, AADC is also expressed in non-dopaminergic neurons and peripheral tissues, which is why L-DOPA must be co-administered with peripheral AADC inhibitors such as carbidopa or benserazide to prevent premature conversion and systemic side effects (LeWitt and Fahn, 2016). Moreover, pyridoxal-5′-phosphate, the active form of vitamin B6, acts as a cofactor for AADC, and its levels must be monitored to maintain optimal drug efficacy.

Despite the progressive degeneration of dopaminergic neurons in PD, L-DOPA retains efficacy because surviving non-neuronal cells can continue converting L-DOPA to dopamine. Studies suggest that L-DOPA may act as a neurotransmitter in denervated striatal tissue, influencing therapeutic responses (Cotzias et al., 1969; Sulzer and Surmeier, 2013).

Once synthesized, dopamine is packaged into synaptic vesicles by VMAT2. This sequestration protects dopamine from cytosolic oxidation and the subsequent formation of reactive oxygen species, a mechanism implicated in PD pathogenesis (Mosharov, 2022; Nepal et al., 2023). Dysregulation of VMAT2 compromises neuronal resilience, and although it is a promising pharmacological target, few agents, such as reserpine and tetrabenazine, modulate it specifically.

Extracellular dopamine is primarily cleared via the DAT, which recycles the neurotransmitter into presynaptic terminals. However, DAT is not entirely selective and can also transport neurotoxins such as 1-methyl-4-phenylpyridinium (MPP^+^), making it both a critical regulator and a potential vector for neurodegeneration (Sulzer and Surmeier, 2013). Transient saturation of DAT during burst firing or neuronal synchronization may enhance dopaminergic signaling but also increase vulnerability under pathological conditions (Dirkx and Bologna, 2022).

Dopamine that escapes re-uptake undergoes enzymatic degradation. Monoamine oxidase (MAO), particularly the MAO-B isoform expressed in glial cells, converts dopamine to 3,4-dihydroxyphenylacetic acid, while catechol-O-methyltransferase (COMT) methylates dopamine to 3-methoxytyramine. The final metabolite, homovanillic acid, is excreted renally (Goldstein and Kopin, 2007). COMT inhibitors such as entacapone and opicapone prolong L-DOPA action and reduce motor fluctuations, although their effects are modest and mostly confined to motor domains (Fabbri et al., 2022).

MAO-B inhibitors, including rasagiline and selegiline, have been shown to reduce oxidative stress and may confer neuroprotective benefits, though their long-term disease-modifying effects remain controversial (Finberg and Rabey, 2016). Nevertheless, their utility in early-stage PD and as adjuncts to L-DOPA in fluctuating patients is well-established in clinical practice.

Recent neuroimaging and functional electrophysiology developments have further refined our understanding of dopaminergic dysfunction in PD. DAT imaging via single-photon emission computed tomography and positron emission tomography has become a standard diagnostic tool to assess nigrostriatal integrity and distinguish PD from atypical parkinsonian syndromes (Dirkx and Bologna, 2022; Hariz and Blomstedt, 2022). Moreover, combining neurochemical profiling with functional data, such as local field potential recordings during DBS, has revealed that motor symptoms correlate with dynamic changes in dopamine-related oscillatory patterns, particularly within beta-band frequencies (Rajamani et al., 2024).

While pharmacological approaches targeting dopamine metabolism have transformed PD care, limitations persist. For example, MAO-B and COMT inhibitors address motor fluctuations but have little impact on axial symptoms or cognitive decline. As the field advances, multimodal strategies integrating precise electrophysiological phenotyping, neurochemical biomarkers, and symptom-specific neuromodulation, such as adaptive DBS, offer a path toward personalized interventions (Malekmohammadi et al., 2022; Roediger et al., 2022; Martinez-Nunez and LeWitt, 2023).

Understanding the complexity of dopamine synthesis, metabolism, and signaling remains pivotal for developing novel therapies in PD. Integrating emerging molecular, electrophysiological, and imaging data will be critical for advancing precision medicine and improving long-term outcomes in this heterogeneous and progressive disorder.

## Dopamine: Neurotransmitter or Neuromodulator?

DA plays a pivotal role in motor regulation, cognitive flexibility, and emotional processing. Its mechanism of action spans both classical neurotransmission and neuromodulation, a duality that underpins its complex involvement in health and disease. Traditionally, neurotransmitters are released into the synaptic cleft in response to presynaptic action potentials, producing rapid and transient effects, typically within milliseconds. Neuromodulators, by contrast, diffuse extrasynaptically, often independent of action potentials, and exert slower, longer-lasting influences across broader neural circuits (Fuxe et al., 2015).

Emerging evidence supports the view that dopamine engages in both forms of chemical signaling. Early electrophysiological studies demonstrated that striatal neurons exhibit excitatory postsynaptic potentials in response to stimulation of nigrostriatal fibers, consistent with classical synaptic transmission (Arias-Carrion et al., 2010). These findings are reinforced by high-resolution imaging showing dopamine release at well-defined synaptic junctions, albeit fewer in number compared to excitatory glutamatergic neurons.

However, dopaminergic terminals also form numerous “open” or synaptic varicosities, sites lacking a distinct postsynaptic density, suggesting an architecture optimized for volume transmission. Each dopaminergic neuron in the substantia nigra projects an extensive arborization, generating up to 500,000 synaptic boutons that allow widespread dopamine diffusion in the extracellular space (Cragg and Greenfield, 1997). The resulting neuromodulatory effects are critical for regulating basal ganglia excitability and for maintaining tonic dopamine tone across target structures.

This capacity for extrasynaptic diffusion is supported by physiological studies showing that dopamine receptor agonists can exert prolonged behavioral effects without action potential-driven release, likely reflecting receptor activation by ambient dopamine levels. Autoregulatory mechanisms, mediated by D2 autoreceptors and DAT, further stabilize extracellular dopamine, enabling consistent neuromodulatory signaling (Nepal et al., 2023).

From a therapeutic perspective, this dual role is exemplified by L-DOPA, the dopamine precursor used as first-line treatment in PD. While some of its efficacy derives from the restoration of phasic synaptic release in residual dopaminergic neurons, much of its benefit may arise from conversion to dopamine in non-neuronal cells, including serotonergic terminals and glia, facilitating extrasynaptic neuromodulation (Cenci, 2014). This mechanism may help explain the robust clinical response to L-DOPA even in advanced stages of PD when nigrostriatal neurons are severely depleted.

The neuromodulatory effects of dopamine also extend beyond the basal ganglia. Dopamine critically modulates hippocampal synaptic plasticity, influencing spatial learning, long-term memory, and behavioral reinforcement processes (Speranza et al., 2021). This broader role is particularly relevant in PD, where dopaminergic loss affects both motor function and non-motor domains such as mood, motivation, and cognition.

Contemporary research supports a model in which dopamine acts as a fast-acting neurotransmitter for precise temporal control and a slow-acting neuromodulator for broader contextual regulation of network dynamics. The interplay between these signaling modes is context-dependent and may be disrupted in disease states, contributing to the multifaceted symptoms of PD and related disorders.

Future treatment strategies may benefit from targeting specific dopaminergic signaling pathways, phasic or tonic, using receptor subtype-selective drugs or adaptive deep brain stimulation protocols that modulate dopamine-linked oscillatory networks (Martinez-Nunez and LeWitt, 2023; Rajamani et al., 2024). Such approaches could more precisely restore physiological dopamine function, improving both motor and non-motor outcomes in PD.

## Autoregulation of Dopaminergic Synapses

Dopaminergic transmission demonstrates remarkable functional stability, maintained by various autoregulatory mechanisms that stabilize extracellular dopamine levels even after the degeneration of some nigrostriatal neurons. These mechanisms may also underlie side effects associated with drugs acting on dopaminergic synapses, such as dyskinesias in PD patients treated with L-DOPA or tardive dyskinesia in patients with schizophrenia treated with neuroleptics. This section discusses critical dopaminergic compensatory mechanisms and their potential implications for PD treatments.

### Presynaptic regulation

Presynaptic regulation of dopaminergic transmission primarily involves control over dopamine synthesis and release, and the mechanisms of such regulation can be described as follows. At the presynaptic terminal, the enzymatic function of TH mediates the conversion of the precursor tyrosine to L-DOPA, where TH participates, the rate-limiting enzyme in dopamine synthesis (Smith et al., 1998; Mulvihill, 2019). Subsequently, L-DOPA is transformed into dopamine via the aromatic L-amino acid decarboxylase enzyme (Mulvihill, 2019). Hence, TH is central to many presynaptic autoregulatory processes. TH activity can be modified by phosphorylation (which enhances activity) and dephosphorylation (Dunkley et al., 2004). Key factors influencing TH activity include (1) intracellular calcium concentration, which fluctuates with action potential frequency; (2) activation of the adenylate cyclase/protein kinase system, which is coupled to presynaptic receptors; and (3) local dopamine concentration, which modulates TH activity through feedback inhibition (Dickson and Briggs, 2013; Bueno-Carrasco et al., 2022).

TH can be inhibited by dopamine and alpha-synuclein (Mor et al., 2017). Dopamine inhibits TH by competing with tetrahydrobiopterin (BH4) for the catalytic binding site (Fanet et al., 2021), decreasing the affinity of the bound dopamine. TH can also be inhibited by excess BH4 (Alterio et al., 1998; Dunkley et al., 2004) and the substrate L-tyrosine (Dunkley et al., 2004). Mulvihill (2019) clearly illustrates the role of the DAT and VMAT2 transporter proteins governing the presynaptic vesicular release of dopamine. The functional interaction between these transporter proteins modulates the cytosolic and extracellular transmitter concentrations of released dopamine from the presynaptic terminal.

In particular, the balance in the cytosolic concentrations of DAT and VMAT2 proteins is crucial in determining the net output of extracellular dopamine released from the presynaptic terminals. Elevated concentration of DAT relative to VMAT2 proteins could lead to lower extracellular dopamine; conversely, elevated levels of VMAT2 relative to DAT could produce higher levels of extracellular dopamine (Mulvihill, 2019).

Other forms of presynaptic regulation of dopaminergic transmission are due to the activation of D2 and D3 dopamine receptors. Dopamine release can be inhibited by the activation of D3 receptors (Gainetdinov et al., 1996). Similarly, Benoit-Marand et al. (2001) found that mice lacking D2 receptors exhibited no inhibition of dopamine release, thus demonstrating the mechanistic involvement of D2 receptors in such inhibition. These findings were consistent with the pioneering observations by Tang et al. (1994), who found that dopamine D2 and D3 receptors inhibit dopamine release in a dopaminergic cell line.

A recent study by Wang et al. (2025) demonstrated that PD128907, a selective dopamine D3 receptor agonist, induced dyskinesia in an animal model of Parkinson’s disease. However, PG01037, a selective dopamine D3 receptor antagonist, attenuated this effect. Similarly, de Boer-Dennert et al. (1997) found that PD128907 decreased the locomotor activity and the extracellular dopamine levels in the striatum. Both studies align with previous findings demonstrating that D2 or D3 dopamine receptor antagonists increase striatal dopamine release *in vivo* in freely moving rats and enhance motor activity (Sotnikova et al., 2005; Rodriguez-Sanchez et al., 2019). These results suggest that selective D3 receptor antagonists may be potential therapeutic targets for managing L-DOPA-induced dyskinesias in PD. However, dopamine regulation involves multiple mechanisms beyond receptor antagonism, including presynaptic autoregulation, metabolic processing, and synaptic plasticity, all of which warrant further investigation. While clinical trials are needed to determine the efficacy of combined D2/D3 receptor antagonism in PD treatment, caution must be exercised given the well-documented adverse effects associated with dopamine receptor modulation, particularly those observed with dopamine receptor agonists (Fusaroli et al., 2024).

### Postsynaptic regulation: Trisynaptic inputs and optogenetic modulation

Postsynaptic regulation of dopamine signaling in the striatum is a complex and dynamic process involving receptor density, intracellular signaling cascades, and synaptic integration of multiple afferents. Medium spiny neurons (MSNs), the principal projection neurons of the striatum, serve as the main integrative hub for converging glutamatergic inputs from the cortex and thalamus and dopaminergic input from the substantia nigra pars compacta. This trisynaptic architecture underpins basal ganglia output and motor control (Gerfen et al., 1990; Gangarossa et al., 2013; Hintiryan et al., 2016).

In PD, the loss of dopaminergic input triggers compensatory upregulation of postsynaptic dopamine receptors, particularly D1 and D2 receptors expressed by MSNs in the direct and indirect pathways, respectively (Missale et al., 1998). This receptor supersensitivity, denervation hypersensitivity, amplifies responsiveness to dopamine agonists and may contribute to motor complications, such as dyskinesias, especially after prolonged L-DOPA exposure (Tang et al., 1994). Notably, such plasticity occurs at the receptor expression level and through downstream modulation of intracellular pathways, including kinases, phosphatases, and transcription factors (Gurevich et al., 2016).

Beyond dopaminergic signaling, MSNs are modulated by adenosine and glutamate receptors, which fine-tune their excitability. The interaction between A2A and D2 receptors, particularly in indirect pathway neurons, forms functional heteromers implicated in motor control. A2A receptor antagonists are under investigation to reduce L-DOPA-induced motor fluctuations (Beaulieu and Gainetdinov, 2011). Similarly, NMDA receptor-dependent glutamatergic plasticity can influence dopaminergic signaling, positioning glutamate modulators as potential therapeutic targets.

Recent optogenetic studies have transformed our understanding of postsynaptic regulation by enabling precise, cell-type-specific control of basal ganglia circuits. Selective activation of D1-expressing direct pathway MSNs enhances movement, whereas stimulation of D2-expressing indirect pathway MSNs induces hypokinesia (Kravitz et al., 2010). These findings provide causal evidence for the classical model of basal ganglia circuitry and highlight the potential of targeted modulation in reversing motor deficits. A further work by Roseberry et al. (2016) mapped distinct basal ganglia outputs to brainstem locomotor centers, refining our understanding of how striatal subcircuits influence specific aspects of motor control.

Optogenetics has also been used to probe pallidothalamic loops. A recent study demonstrated that selective stimulation of the entopeduncular nucleus, the rodent analog of the human globus pallidus internus, ameliorated motor symptoms in a rat model of parkinsonian bradykinesia by dampening abnormal beta oscillations in thalamocortical pathways (Jackson et al., 2025). This represents a significant advance, suggesting that motor symptoms in PD can be alleviated by tuning activity in precise subcircuits rather than solely relying on global stimulation of basal ganglia output structures.

Emerging models of PD pathophysiology have moved beyond traditional direct–indirect pathway dichotomies, incorporating systems-level disruptions in motor code transmission and entropy regulation (Williams, 2023). These models highlight that the core dysfunction in PD may stem from impaired integration across cortical, thalamic, and nigral inputs to MSNs rather than simple alterations in firing rates. Comparative and evolutionary studies suggest that the basal ganglia’s conserved blueprint, preserved over 500 million years, may be especially susceptible to failure under the computational demands of the expanded human cortex (Grillner and Robertson, 2016; Diederich et al., 2019).

These insights underscore the relevance of both synaptic and extrasynaptic mechanisms in striatal output regulation. As optogenetic tools evolve in animal models (Krut et al., 2024), and ethical and legal questions about this technology are answered (Faltus et al., 2023), the possibility of circuit-specific neuromodulation tailored to a patient’s symptom profile becomes increasingly plausible. Future strategies integrating receptor subtype modulation, activity-dependent plasticity, and trisynaptic circuit integrity may transform the treatment landscape for PD, offering precision interventions with improved safety and efficacy.

## Compensatory Mechanisms in Parkinson’s Disease

Compensatory mechanisms in PD include enhanced dopaminergic metabolism with elevated TH activity that increases dopamine release and turnover, reduced re-uptake by DAT, alterations in D2 receptors, and dopamine diffusion with passive stabilization (Blesa et al., 2017). Compensatory mechanisms in PD are highly effective, often preventing the onset of secondary motor symptoms until dopaminergic degeneration exceeds 50%–60% of neurons (Fearnley and Lees, 1991) or 70% of striatal dopaminergic innervation (Hartmann, 2004).

When motor symptoms manifest, these compensatory processes typically operate at maximum capacity and can no longer offset the dopaminergic deficit. However, it has been suggested that these compensatory mechanisms could also behave like a double sword, damaging the remaining neurons more and playing adverse roles in disease progression (Blesa et al., 2017, 2022).

Dopamine receptor agonists specifically target denervated cells, unlike agents such as amphetamines or cocaine, which primarily affect intact systems. In contrast, L-DOPA leverages both presynaptic and postsynaptic compensatory mechanisms (Blesa et al., 2017, 2022). As compensatory capacity diminishes over time, higher drug doses become necessary to maintain therapeutic effectiveness, which can negatively impact healthy neurons. This escalation in dosage can lead to adverse effects that eventually limit the efficacy of dopaminergic therapies. Although “drug holidays” were previously used to restore treatment efficacy, they are now less common due to severe complications during drug breaks and only temporary recovery of therapeutic benefits (LeWitt and Fahn, 2016).

### Long-term complications and dyskinesias

Compensatory mechanisms can contribute to long-term complications such as dyskinesias. Research suggests that “pulsatile” stimulation of dopaminergic transmission by short-acting drugs affects future treatment responses (Olanow et al., 2006). Continuous drug administration may lead to tolerance, while drug-free intervals between doses can cause sensitization. This sensitization may occur due to sustained inhibition of dopamine synthesis and release, triggering postsynaptic compensatory mechanisms that lead to dyskinetic reactions (Calabresi et al., 2008). Treatments aim to achieve “continuous dopaminergic stimulation” to prevent complications and avoid pulsatile effects (Jenner, 2008).

As PD progresses, other challenges appear, including neuronal loss and deficits in drug absorption. Therapy using continuous dopaminergic stimulation has also been applied to address these issues. These include treatments like subcutaneous apomorphine and L-DOPA-carbidopa intestinal gels, which steadily deliver the medication. These interventions activate steady dopamine receptors, reducing motor and non-motor complications like dyskinesias and fluctuations. Infusion therapies are now considered part of the care standard for advanced PD, though access remains a limitation and should be individualized based on patient needs (Pirtošek et al., 2023).

In MPTP primate models, administering small doses of L-DOPA alongside the COMT inhibitor entacapone reduced motor fluctuations and dyskinesias. Combining L-DOPA and entacapone with dopamine agonists further decreased dyskinesia. The results suggest that using L-DOPA and entacapone from the start or as an adjunct therapy in PD could lower the risk of motor complications, warranting clinical trials to confirm these findings in humans (Jenner, 2004).

## Dopaminergic Modulation of Striatal Circuits: Beyond the Classic Pathways

MSNs, first described by Ramón y Cajal, comprise approximately 95%–96% of all neurons in the primate striatum (Yelnik et al., 1991). These GABAergic projection neurons integrate excitatory inputs from the cortex and thalamus and modulatory input from dopaminergic neurons in the SNpc (Surmeier et al., 2007). Each MSN possesses around 30,000 dendritic spines, and a single neuron may receive synaptic input from thousands of cortical neurons. Despite this convergence, MSNs remain mostly quiescent and fire only when a critical threshold of synchronous excitatory input is reached, a property reinforced by intense lateral inhibition from neighboring MSNs (Gertler et al., 2008).

MSNs are subdivided into two main populations based on their receptor expression and projection targets: those forming the direct pathway express D1 dopamine receptors and project to the internal segment of the globus pallidus internus, and SNpr, while those forming the indirect pathway express D2 receptors and project first to the external globus pallidus, and then through the subthalamic nucleus to the globus pallidus internus/SNpr (Smith et al., 1998; Gerfen and Surmeier, 2011; Lanciego et al., 2012). These circuits form the foundation of basal ganglia output pathways that modulate movement via GABAergic projections to the motor thalamus and subsequent cortical feedback loops.

However, recent findings suggest that this binary model of striatal output oversimplifies the true complexity of basal ganglia dynamics. Advanced tracing, optogenetics, and *in vivo* imaging have revealed overlapping and parallel circuits, sometimes involving the same MSNs, that participate in both motor and cognitive domains (Williams, 2023). MSNs are now understood to operate within broader, trisynaptic loops involving cortical, thalamic, and midbrain afferents that simultaneously converge on individual MSNs, refining motor selection and inhibition with millisecond-level precision (Gangarossa et al., 2013; Hintiryan et al., 2016).

Recent optogenetic studies have provided compelling evidence for pathway-specific modulation. Selective stimulation of direct pathway MSNs alleviates bradykinesia in parkinsonian models, while indirect pathway MSNs exacerbate motor inhibition (Kravitz et al., 2010). Furthermore, using optogenetic methods, cell-type-specific control of pallidothalamic circuits has modulated motor behavior in rats, offering a proof-of-concept for circuit-specific neuromodulation (Jackson et al., 2025). These findings have helped shift the field toward a more network-centric model, where basal ganglia output is shaped by the context-dependent integration of multi-nodal inputs rather than simple linear pathways (Grillner and Robertson, 2016; Williams, 2023).

Although MSNs dominate the striatal landscape, interneurons, which comprise only 3%–4% of the striatal neuronal population, play critical roles in shaping MSN excitability and output. Among these, cholinergic interneurons, or tonically active neurons, are particularly well-studied. These large aspiny neurons receive dopaminergic input and respond to reward-related stimuli with transient pauses in activity. This pause is abolished by D2 receptor antagonism or dopaminergic denervation, highlighting the functional integration between dopamine and acetylcholine signaling in the striatum (Aosaki et al., 1994; Raz et al., 1996). Fast-spiking GABAergic interneurons also exert feedforward inhibition on MSNs, although their role in PD pathophysiology remains incomplete (Koos et al., 2004).

Human neuroimaging studies have further challenged the classical model by identifying distinct circuits within the basal ganglia–thalamo-cortical loops selectively altered in PD (Postuma and Dagher, 2006; McGregor and Nelson, 2019). These circuits show differential vulnerability to dopaminergic depletion and are implicated not only in motor symptoms but also in non-motor manifestations of PD, including executive dysfunction, apathy, and mood disorders (Nambu, 2008; Diederich et al., 2019).

These findings support an expanded framework in which MSNs function as integrative nodes within dynamic, plastic, and partially overlapping circuits. These circuits are modulated by dopamine, acetylcholine, and glutamate and shaped by the anatomical convergence of inputs from multiple brain regions. As the field moves beyond rigid dichotomies of direct and indirect pathways, there is growing emphasis on pathway-specific interventions, including optogenetic and closed-loop stimulation strategies, that can precisely modulate striatal output in a context-dependent and symptom-specific manner.

## Vulnerability of Dopaminergic Neurons and Parkinson’s Disease

The selective vulnerability of dopaminergic neurons within the SNpc remains a defining yet incompletely understood PD feature. Structural, neurochemical, and genetic factors contribute to the degeneration of these neurons, resulting in hallmark motor symptoms such as tremor, rigidity, and bradykinesia, which typically emerge only after the loss of 40%–60% of SNpc neurons and a 60%–70% depletion of striatal dopamine levels (Dauer and Przedborski, 2003; Blesa et al., 2022).

### Spatial and molecular heterogeneity in dopaminergic vulnerability

Recent advances in single-cell and spatial transcriptomics have provided compelling evidence that dopaminergic neurons within the SNpc are not a homogenous population but instead comprise distinct molecular subtypes with differential susceptibility to degeneration (Kamath et al., 2022; Wang et al., 2025). Notably, a subset of DA neurons expressing AGTR1 and enriched in TP53 and NR2F2 transcriptional signatures was found to be spatially restricted to the ventral SNpc and disproportionately affected in PD (Kamath et al., 2022; Wang et al., 2025). This population also showed heritable enrichment for PD-associated genetic risk, underscoring a cell-intrinsic vulnerability.

A parallel study by Wang et al. (2024) identified additional molecularly distinct neuron clusters within the SNpc using snRNA-seq. Among these, RIT2-enriched neurons, a population previously associated with PD risk in genome-wide association studies, also demonstrated marked susceptibility to neurodegeneration. These findings refine the longstanding dorsoventral gradient of vulnerability by linking it to specific transcriptional programs and risk alleles (Yamada et al., 1990; Damier et al., 1999).

### Dopaminergic metabolism and oxidative stress

Oxidative stress, driven partly by dopamine metabolism, plays a central role in SNpc degeneration. Dopaminergic neurons rely heavily on mitochondrial respiration, particularly sensitive to mitochondrial dysfunction and reactive oxygen species (ROS). DA not sequestered into vesicles by VMAT2 undergoes cytosolic auto-oxidation, forming toxic quinones and generating ROS (Segura-Aguilar et al., 2014; Mosharov, 2022). A high DAT/VMAT2 ratio facilitates cytosolic dopamine accumulation, exacerbating oxidative stress (Sulzer and Surmeier, 2013; Mosharov, 2022).

Mitochondrial DNA (mtDNA) in SNpc neurons is especially vulnerable due to its proximity to ROS-producing complex I, the absence of protective histones, and inefficient repair mechanisms (Tresse et al., 2023). Age-related declines in mtDNA fidelity and antioxidant capacity, reflected in reduced superoxide dismutase levels in PD plasma, compound this vulnerability (Chidambaram et al., 2024).

### Transcriptional regulation and cellular stress pathways

Recent work has illuminated how molecular programs within vulnerable neuron subtypes predispose them to degeneration. For instance, NR2F2, a transcription factor implicated in mitochondrial dysfunction and apoptosis, is selectively upregulated in AGTR1-positive neurons, as is TP53, which links PD to other neurodegenerative conditions (Kamath et al., 2022). These findings suggest that PD risk is amplified within a transcriptionally defined neuron subtype due to inherent stress-response failures.

### Neuroinflammation and glial modulation

Beyond neurons, neuroinflammation and glial cell dysfunction play key roles in PD pathology. Microglial activation and astrocytic responses, often marked by ICAM-1 and TSPO binding, have been observed in PD brains, although their extent remains debated (Balzano et al., 2024; Wang et al., 2024). Notably, expression of SNCA (encoding α-synuclein) was found to be reduced in dopaminergic and glutamatergic neurons but elevated in microglia and oligodendrocytes in PD, suggesting cell-type-specific shifts in α-synuclein burden (Wang et al., 2024).

Moreover, peripheral inflammatory markers, including plasma interleukin-1β and NLRP3 (NOD-, LRR- and pyrin domain-containing protein 3) inflammasome components, have emerged as potential biomarkers of early nigral inflammation and correlate with motor severity in PD (Fan et al., 2020; Anderson et al., 2023; Chidambaram et al., 2024).

### α-Synuclein and iron

The misfolding and aggregation of α-synuclein, encoded by the SNCA gene, disrupts vesicular trafficking, mitochondrial function, and proteostasis, particularly in neurons with extensive axonal arborization like those in the SNpc (Burbulla et al., 2017; Padilla-Godinez et al., 2025). Its interactions with dopamine oxidation products yield soluble oligomers that interfere with cellular machinery, contributing to PD pathogenesis (Padilla-Godinez et al., 2025).

Iron accumulation in the SNpc further enhances α-synuclein aggregation and ROS generation, forming a pathological feedback loop that accelerates neurodegeneration. Iron-mediated post-translational modifications of α-synuclein increase its propensity to form toxic oligomers, and this iron burden may be amplified by neuronal morphology and energy demands (Uversky et al., 2001; Giguere et al., 2019).

### Towards an integrated model of selective vulnerability

These insights support a convergent model in which mitochondrial stress, transcriptional dysregulation, impaired dopamine handling, and neuroinflammation selectively impair molecularly defined neuron subtypes within the SNpc. Single-cell profiling efforts now enable the identification of such subtypes, such as AGTR1^+^/SOX6^+^ or RIT2-enriched neurons, and provide a roadmap for precision targeting of neuroprotective therapies (Kamath et al., 2022; Wang et al., 2024).

Therapeutic strategies to modulate oxidative stress, iron load, α-synuclein accumulation, and inflammation will need to incorporate cell-type-specific vulnerabilities. Longitudinal studies with molecular imaging and plasma biomarkers may help refine early diagnostic criteria and guide targeted interventions in PD.

## Discussion and Future Perspectives

**Figure 1:**
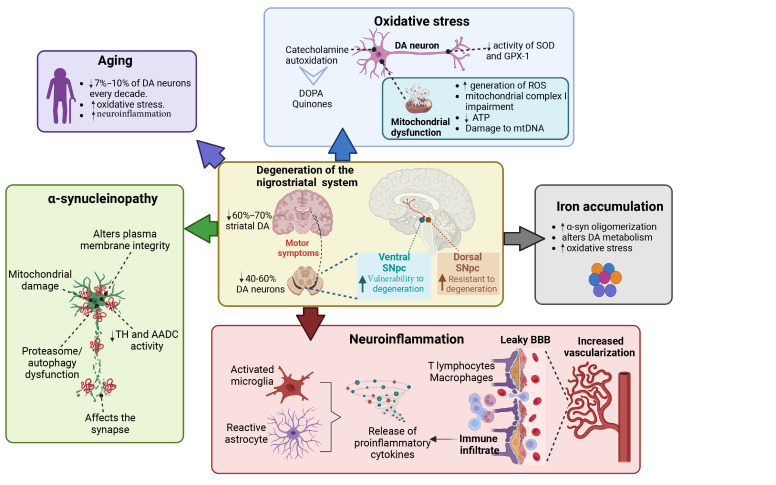
Mechanisms driving nigrostriatal degeneration in Parkinson’s disease. This figure illustrates the multifactorial mechanisms contributing to the selective vulnerability and progressive degeneration of nigrostriatal DAergic neurons in Parkinson’s disease. Aging promotes oxidative stress and neuroinflammation, contributing to the loss of approximately 7%–10% of DA neurons per decade. Neurons in the ventral SNpc are especially susceptible, whereas those in the dorsal SNpc exhibit greater resilience. Mitochondrial dysfunction — including ROS production, impaired complex I activity, ATP depletion, and mtDNA damage — undermines neuronal viability. Iron accumulation facilitates the aggregation of α-syn and promotes Fenton chemistry, which enhances oxidative damage. Cytosolic dopamine, when unbuffered by vesicular monoamine transporter 2, undergoes autoxidation to form toxic DOPA quinones, destabilizing dopamine homeostasis. Misfolded α-syn impairs proteasomal and autophagic clearance pathways, reducing the function of TH and AADC, thereby impairing dopamine synthesis. As degeneration advances, 40%–60% of SNpc DA neurons are lost, and striatal dopamine levels decline by 60%–70%, triggering motor symptoms. Neuroinflammation — characterized by activated microglia, reactive astrocytes, peripheral immune cell infiltration (T lymphocytes, macrophages), increased vascularization, and BBB breakdown — further amplifies neurodegenerative cascades. Conceptual design by the authors, based on integrated findings from Kamath et al. (2022), Wang et al. (2024), and others cited in the manuscript. ↑ Indicates increased expression or activity; ↓ indicates decreased expression or function. Created with BioRender.com. AADC: Aromatic L-amino acid decarboxylase; α-syn: alpha-synuclein; ATP: adenosine triphosphate; BBB: blood–brain barrier; DA: dopamine; DAergic: dopaminergic; DOPA: dihydroxyphenylalanine; GPX-1: glutathione peroxidase 1; mtDNA: mitochondrial DNA; ROS: reactive oxygen species; SNpc: substantia nigra pars compacta; SOD: superoxide dismutase; TH: tyrosine hydroxylase.

The nigrostriatal dopaminergic system regulates motor, cognitive, and emotional functions. Its progressive degeneration in PD results in a cascade of disabling symptoms. This degeneration is driven by a complex interplay of molecular, cellular, and systemic factors that confer selective vulnerability to dopaminergic neurons in SNpc (**Figure 1** and **Table 1**). A growing body of evidence supports the need to reframe PD not only as a disease of dopamine deficiency but also as one of neuronal subtype-specific degeneration requiring tailored neuroprotective strategies (Kamath et al., 2022; Stocchi et al., 2024).

**Table 1:** Dopaminergic vulnerability of nigrostriatal neurons

Feature	Description
Anatomical disproportion	SNpc volume (approximately 0.056 cm³) is approximately 0.9% of striatal volume (6.2 cm³) → a small region governs extensive motor function via long-range projections.
Extensive axonal arborisation	A single neuron forms up to 1,000,000 synapses → high metabolic and proteostatic burden predisposes to oxidative stress.
Dorsoventral gradient	Ventral SNpc neurons are more susceptible than dorsal → aligns with the loss of SOX6^+^/AGTR1^+^ subtypes identified in human PD brains (Kamath et al., 2022; Wang et al., 2024).
Electrophysiological properties	Intrinsic pacemaking (0.5–10 Hz) and burst firing patterns → sustained calcium influx increases mitochondrial load and ROS generation.
Neurochemical diversity	Subtypes express different combinations of TH, ALDH1A1, Girk2, DAT, and VMAT2 → each confers distinct vulnerability/resilience profiles.
Transcriptional identity	Single-nucleus RNA-seq reveals molecularly distinct populations (e.g., RIT2^+^, CALB1_GEM) with differential susceptibility → molecular markers predict degeneration.
Neurotransmitter dynamics	Dopamine homeostasis regulated by DAT, VMAT2, D2/D3 autoreceptors → imbalanced exacerbate oxidative stress and synaptic dysfunction.
Glutamatergic input	Trisynaptic input from the cortex, thalamus, and midbrain regulates MSN activity → altered glutamate transporter EAAT2 expression in LRRK2 mutation carriers.
Neuroinflammation	Activation of microglia and astrocytes promotes IL-1β, TNF-α, and NLRP3-driven responses → inflammasome activity linked to disease severity (Fan et al., 2020).
Mitochondrial dysfunction	Reduced ATP production, mtDNA damage, and Complex I impairment → impaired vesicular packaging of DA leads to cytosolic DA and ROS.
α-Synuclein aggregation	Aggregated α-synuclein disrupts vesicle trafficking and proteostasis → Interaction with dopamine and iron fosters toxic oligomers.
Iron accumulation	Iron deposits in SNpc → amplify α-synuclein misfolding and mitochondrial damage via Fenton chemistry.
Vascular and immune factors	Increased vascularization in ventral SNpc permits the entry of immune cells and toxins → promoted degeneration (Balzano et al., 2024).
Aging-related decline	Physiological dopamine neuron attrition (7%–10% per decade) → accelerated in PD due to increased mitochondrial and inflammatory burden.

AGTR1: Angiotensin II receptor type 1; ALDH1A1: aldehyde dehydrogenase 1 family member A1; ATP: adenosine triphosphate; DA: dopamine; DAT: dopamine transporter; EAAT2: excitatory amino acid transporter 2; Girk2: G-protein inward rectifier potassium channel 2; IL-1β: interleukin-1 beta; LRRK2: leucine-rich repeat kinase 2 gene; mtDNA: mitochondrial DNA; NLRP3: NOD-, LRR- and pyrin domain-containing protein 3; PD: Parkinson’s disease; RIT2: Ras-like without CAAX 2; ROS: reactive oxygen species; SNpc: substantia nigra pars compacta; SOX6: SRY-box transcription factor 6; TH: tyrosine hydroxylase; TNF-α: tumor necrosis factor alpha; VMAT2: vesicular monoamine transporter 2.

Four main factors have been consistently implicated in dopaminergic vulnerability: oxidative stress, mitochondrial dysfunction, neuroinflammation, and α-synuclein pathology. Oxidative stress arises from dopamine metabolism, pacemaking activity, and extensive axonal arborization, which impose bioenergetic strain and promote ROS generation. When ROS exceeds the buffering capacity of protective enzymes such as superoxide dismutase and glutathione peroxidase, cellular structures are damaged, and dopaminergic neurons undergo degeneration (Mosharov et al., 2009; Blesa et al., 2022; Mosharov, 2022). Cytosolic accumulation of dopamine, driven by an imbalance between DAT and VMAT2, has been shown to contribute to ROS generation and cytotoxic quinone formation.

Mitochondrial dysfunction is a converging node for multiple stressors. The high energy demands of SNpc neurons, inefficient DNA repair, and the absence of histones in mtDNA make these cells susceptible to ROS-induced damage (Tresse et al., 2023). Single-cell transcriptomic studies confirm that vulnerable neuron subtypes, such as AGTR1^+^/SOX6^+^ populations, exhibit mitochondrial stress signatures prior to overt cell loss, indicating early windows for intervention (Kamath et al., 2022; Wang et al., 2025).

Neuroinflammation, once considered a secondary phenomenon, now actively contributes to disease progression. Astrocyte and microglial activation precede dopaminergic degeneration, and PD patients show peripheral immune activation and increased expression of inflammasome-related markers such as NLRP3 and interleukin-1β, which correlate with disease severity (Fan et al., 2020; Anderson et al., 2023). New findings suggest that increased vascularization in the ventral SNpc may permit immune cell infiltration and toxin entry, exacerbating inflammatory cascades (Balzano et al., 2024).

The central pathological hallmark of PD, α-synuclein aggregation, intersects with all these mechanisms. Misfolded α-synuclein interacts with dopamine and iron to generate toxic oligomers, disrupts mitochondrial function, and impairs autophagy and proteostasis (Uversky et al., 2001). RIT2-enriched neurons, which express high levels of α-synuclein-interacting genes and exhibit increased vulnerability in postmortem and in vitro models, exemplify how molecular identity governs degeneration risk (Wang et al., 2024, 2025).

Beyond these four pillars, recent single-cell and spatial transcriptomic technologies have revealed that the SNpc is composed of at least ten molecularly distinct dopaminergic subtypes, each with specific developmental origins, gene expression profiles, and resilience to degeneration (Kamath et al., 2022; Wang et al., 2024). For instance, SOX6^+^/AGTR1^+^ neurons are preferentially depleted in PD and show upregulation of TP53 and NR2F2, which are linked to apoptosis and mitochondrial stress. This refined classification challenges the notion of a homogeneous nigral population and opens the door to precision-targeted therapies.

New therapeutic avenues include neuroprotective strategies aimed at oxidative stress and mitochondrial support (Keethedeth and Anantha Shenoi, 2025), anti-inflammatory agents targeting inflammasomes and cytokines (Dzamko, 2023), and emerging interest in gut–brain interactions that modulate neuroimmune signaling (Riegelman et al., 2024). Gene editing tools such as CRISPR-Cas9, alongside cell-based approaches using induced pluripotent stem cells, are being actively explored to restore dopaminergic function, though challenges such as immune rejection and functional integration remain (Mansour et al., 2023).

Biomarker development is critical for early diagnosis and therapeutic monitoring. Neuroimaging tracers such as ACI-12589PET and 18F-F0502BPET, which selectively bind to α-synuclein, represent significant advances in visualizing pathology *in vivo* (Smith et al., 2023). Fluid-based assays, including seed amplification techniques for α-synuclein misfolding, show promise in identifying prodromal disease and distinguishing PD from other synucleinopathies (Kuang et al., 2024). Integrating these tools into longitudinal studies could facilitate patient stratification and therapeutic timing.

An integrative research framework that aligns genetic risk loci with molecular neuron identity is beginning to emerge. Data from human snRNA-seq studies now inform genome-wide association studies interpretation, prioritizing genes that are actively expressed in vulnerable SNpc subtypes (Carmichael et al., 2021; Kamath et al., 2022; Wang et al., 2025). Such strategies enable more rational drug development and targeted neuroprotection.

In clinical settings, combined approaches involving continuous dopaminergic stimulation, adaptive deep brain stimulation, and personalized pharmacogenetics may minimize motor fluctuations and maximize quality of life. However, to meaningfully alter the disease course, future therapies must address the early, subtype-specific mechanisms of vulnerability that precede widespread cell loss.

In conclusion, while tremendous strides have been made in characterizing the neurobiology of PD, the translation of these insights into effective interventions remains incomplete. Understanding the selective vulnerability of dopaminergic subtypes and developing subtype-specific neuroprotective strategies will be crucial for achieving meaningful disease modification. A multidisciplinary and collaborative approach that leverages molecular profiling, biomarker discovery, and personalized therapeutics holds the greatest promise for transforming PD care in the years ahead.

OPEN PEER REVIEW REPORT 1

## Data Availability

*Not applicable*.
