# Supplementary material for: Nigrostriatal dopaminergic vulnerability in Parkinson’s disease: Neuroprotective strategies
Source: Neural Regen Res. 2025 Aug 13;21(8):3378–86. doi: 10.4103/NRR.NRR-D-25-00380 (PMC13452663; doi:10.4103/NRR.NRR-D-25-00380)
Supplement: Supplementary file 1 [file NRR-21-3378_Suppl1.pdf]

OPEN PEER REVIEW REPORT 1

Name of journal: Neural Regeneration Research
Manuscript NO: NRR-D-25-00380
Title: Neurobiology of nigrostriatal dopaminergic vulnerability in Parkinson's disease: Neuroprotective strategies
Reviewer's Name: Grazyna Niewiadomska
Reviewer's country: Poland

COMMENTS TO AUTHORS

Overall evaluation on article quality:

The manuscript submitted for review entitled: "Neurobiology of Nigrostriatal Dopaminergic Vulnerability in Parkinson's Disease: Neuroprotective strategies" is written in a manner that is both accurate and precise, and is characterised by a clearly articulated thesis and a comprehensive understanding of the subject matter. The promotion of a deeper understanding of the mechanisms underlying vulnerability of the dopaminergic nigrostriatal system is imperative for the development of novel neuroprotective treatments for Parkinson's disease, representing a crucial and urgent strategy in clinical management. I have no major objections to the reviewed manuscript. The paper is written in a polished style, with a sufficient selection of studies discussed and the manuscript is also very carefully prepared from an editorial point of view.

The following minor comments are offered for consideration by the authors:

1. The last paragraph of section 2.1 Electrophysiological characteristics:
This paragraph is relatively concise and lacks detailed knowledge and factual evidence regarding the electrophysiological correlates of signalling in the nigrostriatal system. It is imperative to ascertain the handle points for the application of potential novel diagnostic techniques (which ones?).

2. The third paragraph of section 4.2 Postsynaptic regulation
It is evident that this paragraph is lacking in its consideration of the trisynaptic signalling system that converges on the medium spiny neurons of the striatum (dopaminergic input from substantia nigra, excitatory glutamatergic signalling from the cortex, modulating actions of cholinergic interneurons and fast discharging small GABAergic interneurons). This system is of critical importance in the generation of projection signals to structures that have anatomical connections to the striatum and are significant in the motor control.

And the next paragraph in this section: This paragraph is based on a single cited publication from 2017 and does not provide specific information on which signalling pathways and what motor behaviours are modulated by optogenetic stimulation in specific clinical applications.

3. The second paragraph of section 6. The action of dopaminergic neurons in the striatum
This paragraph suggests a lack of knowledge about the regulation of signalling in the striatum. There are now a number of recent publications on the organisation and function of the direct and indirect pathways of MSN stimulation by dopaminergic neurons located in the substantia nigra.
